# LIVING FOREVERWELL IN RURAL AMERICA: RESULTS FROM A QUALITATIVE EVALUATION OF A YMCA HEALTHY AGING PROGRAM

**DOI:** 10.1093/geroni/igad104.0069

**Published:** 2023-12-21

**Authors:** Rachel Kappel, Alycia Bayne, Shena Popat, Mabel Frank, Seana Hasson, Lindsay Mondick

**Affiliations:** NORC at the University of Chicago, Chicago, Illinois, United States; NORC at the University of Chicago, Chicago, Illinois, United States; NORC at the University of Chicago, Chicago, Illinois, United States; NORC at the University of Chicago, Chicago, Illinois, United States; YMCA of the USA, Chicago, Illinois, United States; YMCA of the USA, Eagan, Minnesota, United States

## Abstract

Older adults benefit from community programs that promote physical and mental health, reduce social isolation, and offer connections to community resources. However, older adults living in rural communities have access to fewer health and social services and experience barriers to participation such as access to transportation. Recognizing these challenges, the YMCA of the USA (Y-USA) expanded ForeverWell, a healthy aging model, to 17 YMCAs (“Ys”) serving adults aged 55+ living in rural areas. Developed by the YMCA of the North in Minnesota, ForeverWell is a flexible set of Y activities focused on five dimensions of healthy aging: mind, body, community, spirit, and nature. The goals of ForeverWell are to improve health and well-being, decrease social isolation, and increase community connections among older adults. NORC at the University of Chicago conducted a qualitative evaluation of ForeverWell’s expansion in 17 communities in Minnesota, North Dakota, and South Dakota. NORC conducted virtual interviews with 25 Y staff members and 24 program participants to identify facilitators, challenges, and outcomes. NORC transcribed interviews and conducted qualitative content analysis to identify themes. This session describes findings from the interviews. A key facilitator of success was the flexibility of the model. Challenges included staff and financial resources, outreach in rural areas, and transportation access. Ys developed and enhanced older adult programming and created new partnerships. Program participants reported improvements in physical and mental health and strong social connections. ForeverWell is a flexible, holistic healthy aging program that shows promise for older adults living in rural communities.

